# Awareness of fetal movements and care package to reduce fetal mortality (AFFIRM): a stepped wedge, cluster-randomised trial

**DOI:** 10.1016/S0140-6736(18)31543-5

**Published:** 2018-11-03

**Authors:** Jane E Norman, Alexander E P Heazell, Aryelly Rodriguez, Christopher J Weir, Sarah J E Stock, Catherine J Calderwood, Sarah Cunningham Burley, J Frederik Frøen, Michael Geary, Fionnuala Breathnach, Alyson Hunter, Fionnuala M McAuliffe, Mary F Higgins, Edile Murdoch, Mary Ross-Davie, Janet Scott, Sonia Whyte

**Affiliations:** a Tommy's Centre for Maternal and Fetal Health, MRC Centre for Reproductive Health, Queen's Medical Research Institute, University of Edinburgh, Edinburgh, UK; b Tommy's Maternal and Fetal Health Research Centre, School of Medical Sciences, Faculty of Biology, Medicine and Health, University of Manchester, Manchester, UK; c St Mary's Hospital, Central Manchester University Hospitals NHS Foundation Trust, Manchester Academic Health Science Centre, Manchester, UK; d Centre for Population Health Sciences, Usher Institute of Population Health, Sciences and Informatics, University of Edinburgh, Edinburgh, UK; e The Scottish Government, Edinburgh, UK; f Global Health Cluster, Division for Health Services, Norwegian Institute of Public Health, Oslo, Norway; g Rotunda Hospital, Dublin, Ireland; h Centre for Fetal Medicine, Royal Maternity Hospital, Belfast, UK; i UCD Perinatal Research Centre, School of Medicine, University College Dublin, National Maternity Hospital, Dublin, Ireland; j Department of Neonatology, Royal Infirmary of Edinburgh, NHS Lothian, Edinburgh, UK; k Royal College of Midwives, Edinburgh, UK; l Sands, Victoria Charity Centre, London, UK

## Abstract

**Background:**

2·6 million pregnancies were estimated to have ended in stillbirth in 2015. The aim of the AFFIRM study was to test the hypothesis that introduction of a reduced fetal movement (RFM), care package for pregnant women and clinicians that increased women's awareness of the need for prompt reporting of RFM and that standardised management, including timely delivery, would alter the incidence of stillbirth.

**Methods:**

This stepped wedge, cluster-randomised trial was done in the UK and Ireland. Participating maternity hospitals were grouped and randomised, using a computer-generated allocation scheme, to one of nine intervention implementation dates (at 3 month intervals). This date was concealed from clusters and the trial team until 3 months before the implementation date. Each participating hospital had three observation periods: a control period from Jan 1, 2014, until randomised date of intervention initiation; a washout period from the implementation date and for 2 months; and the intervention period from the end of the washout period until Dec 31, 2016. Treatment allocation was not concealed from participating women and caregivers. Data were derived from observational maternity data. The primary outcome was incidence of stillbirth. The primary analysis was done according to the intention-to-treat principle, with births analysed according to whether they took place during the control or intervention periods, irrespective of whether the intervention had been implemented as planned. This study is registered with www.ClinicalTrials.gov, number NCT01777022.

**Findings:**

37 hospitals were enrolled in the study. Four hospitals declined participation, and 33 hospitals were randomly assigned to an intervention implementation date. Between Jan 1, 2014, and Dec, 31, 2016, data were collected from 409 175 pregnancies (157 692 deliveries during the control period, 23 623 deliveries in the washout period, and 227 860 deliveries in the intervention period). The incidence of stillbirth was 4·40 per 1000 births during the control period and 4·06 per 1000 births in the intervention period (adjusted odds ratio [aOR] 0·90, 95% CI 0·75–1·07; p=0·23).

**Interpretation:**

The RFM care package did not reduce the risk of stillbirths. The benefits of a policy that promotes awareness of RFM remains unproven.

**Funding:**

Chief Scientist Office, Scottish Government (CZH/4/882), Tommy's Centre for Maternal and Fetal Health, Sands.

## Introduction

Stillbirth is a pervasive problem worldwide. 2·6 million babies were estimated to have died in utero in 2015.^1^ In high-income countries (HICs), one in 113–769 pregnancies end in stillbirth after 28 weeks.^2^ Most stillbirths happen without fetal abnormality or pre-existing risk factors for stillbirth. The six-fold variation in the incidence of stillbirth in HICs, suggests that a large proportion of stillbirths are possibly preventable.^2^

Maternal perception of reduced fetal movement (RFM) has been identified as a potential strategy for stillbirth prevention. The link between RFM and stillbirth (or the causes of stillbirth) is clear. 30–55% of women whose pregnancies end in stillbirth experience RFM in the preceding week.^3^, ^4^ RFM is also associated with fetal growth restriction^5^ and placental abnormalities in pregnancies that do not end in stillbirth.^6^, ^7^ However, RFM is only modestly associated with increased risk of stillbirth (odds ratios [OR] 2·37–14·1),^8^, ^9^ and whether RFM is a symptom of inevitable fetal death or whether it can be used as an alert to prompt action and improve outcome is unclear. In a Cochrane review (dominated by one cluster-randomised trial of 68 000 women allocated to formal kick counting or usual treatment^10^), formal fetal movement counting was concluded to be of uncertain benefit as a test of fetal wellbeing.^11^

Interest in RFM as a stillbirth prevention tool has been renewed in recent years. The arbitrary threshold of normal movements (as used by Grant and colleagues^10^) is widely recognised as unhelpful; rather, the woman's perception of RFM is important.^12^ Crucially, maternal perception of RFM can only improve outcome if action is taken. In Norway, as part of a seminal quality improvement project, a package of care was introduced to raise awareness of the importance of reporting RFM, combined with action to detect babies that are small for gestational age and expedited delivery of babies at risk. The incidence of stillbirth decreased from 3·0 stillbirths per 1000 pregnancies to 2·0 stillbirths per 1000 pregnancies.^13^ Consequently, several groups have prioritised RFM for stillbirth research.^2^, ^14^

**Research in context**

**Evidence before this study**

Awareness of reduced fetal movement (RFM) is promoted to reduce stillbirth, but the evidence to support the effect of RFM awareness is uncertain. We searched PubMed in 2013, and updated the search on March 23, 2018, using the terms “reduced fetal movement” AND “randomised trials” OR “systematic review”. We found one systematic review evaluating routine fetal movement counting compared with mixed or undefined fetal movement counting, but there were no differences in the incidence of stillbirth or perinatal mortality between the groups. All the data to inform this finding came from a single cluster-randomised trial of 68 654 women, with a standard mean difference in incidence of stillbirth per cluster of 0·23 per 1000 (95% CI −0·61 to 1·07), with a trend to higher risk in the intervention clusters.

**Added value of this study**

To the best of our knowledge, this study is the first to combine RFM as an alert with an intervention designed to reduce the risk of stillbirth, and the largest study of fetal movement awareness to date. In a stepped wedge, cluster-randomised trial of 409 175 pregnancies, we showed that a package of care for pregnant women and clinicians to raise awareness of the importance of RFM, combined with a fuller assessment of fetal wellbeing and expedited delivery (where the benefits were likely to outweigh the risks), had no significant effect on the risk of stillbirth. The incidence of stillbirth at or beyond 24 weeks' gestation was 4·06 per 1000 livebirths during the intervention period and 4·40 per 1000 livebirths during the control period (adjusted odds ratio 0·90, 95% CI 0·75–1·07; p=0·232). Our secondary outcomes include a surrogate of stillbirth, the proportion of babies at or below the 10th centile of gestationally adjusted birthweight delivered at 40 weeks or more. This stillbirth surrogate was lower in the intervention group (3461 [1·5%] events of 227 860 births) than the control group (3081 [2·0%] of 157 692; p=0·001). This potential benefit has to be set against the higher frequency of caesarean section (64572 [28·4%] of 227 860 births during the intervention period *vs* 40 231 [25·5%] of 157 692 births during the control period; p<0·001] and induction rates (83 499 [40·7%] of 227 860 *vs* 49 952 [35·9%] of 157 692; p=0·001).

**Implications of all the available evidence**

RFM awareness is not supported by the research to date. Future research should include completion of other ongoing fetal movement awareness studies and a meta-analysis of data from all studies combined. An economic analysis of these data will supply additional evidence on the effectiveness of RFM awareness as a stillbirth reduction strategy, the costs, and any effects of increased rates of intervention. Such evidence will help policy makers make informed decisions about how RFM awareness might fit into a stillbirth reduction strategy.

Evidence-based and accurate information is necessary to improve pregnancy management, which is variable and often suboptimal in women with RFM.^15^, ^16^ Crucially, proof that promoting awareness of the importance of RFM (in combination with appropriate management) has benefits is inadequate. Nevertheless, many health-service providers incorporate fetal movement awareness in their stillbirth reduction strategies. The aim of the AFFIRM study was to formally evaluate, in a randomised trial, a package of care that included awareness-raising of the importance of RFM in pregnant women and clinicians, combined with an improved assessment of fetal wellbeing and expedited delivery where the benefits were likely to outweigh the risks. We tested the hypothesis that the introduction of a RFM care package would alter the incidence of stillbirth.

## Methods

### Study design and participants

This continuous recruitment short exposure, stepped wedged, cluster-randomised trial was done in public maternity hospitals and maternity units in the UK and Ireland. All maternity hospitals in Scotland were expected to join, whereas hospitals in England, Wales, and Ireland joined voluntarily. A complete list of trial investigators and coordinators for the AFFIRM study is provided in the appendix.

Ethics approval was obtained from the Scotland A Research Ethics Committee (Ref 13/SS/0001). The committee agreed that individual patient consent was not required. The trial protocol has been published.^17^

Participating hospitals largely followed a so-called shared care model provided by midwives and obstetricians. All participating hospitals agreed to participate before the study started, and formal written consent was obtained from hospital research and development departments, with input from the lead obstetric clinician in each participating hospital. Data were collected for all women delivering in participating maternity hospitals during the study period. No women were excluded other than those who asked to be withdrawn from routine data collection. Individual women were not asked for consent to treatment allocation.

### Randomisation and masking

Hospitals (each representing a cluster in the design) were grouped before randomisation, each with a sum of about 17 000 deliveries annually, with alignment by geographical location to minimise contamination (appendix).

Clusters were randomised before the beginning of the trial, using a computer-generated allocation scheme, to one of nine intervention implementation dates. This timing was concealed from clusters and the trial team until 3 months before the implementation date. Three to five clusters were randomised at each timepoint. No attempt was made to conceal treatment allocation from women or clinicians.

### Procedures

Each participating hospital had three observation periods: the first from Jan 1, 2014, until randomised date of intervention initiation (the control period); the second from the initiation date and for 2 months (the washout period); and the third from the end of the washout period until Dec 31, 2016 (the intervention period). Comparisons of pregnancy outcomes for births during the control period and the intervention period were used to determine the effectiveness of the intervention.

The trial intervention included an e-learning education package for all clinical staff in participating hospitals about the importance of a recent change in the frequency of fetal movements and how to manage RFM, a leaflet for pregnant women (usually distributed to women at about 20 weeks' gestation). A management plan for identification and delivery of babies at high risk was distributed to hospitals for management of women who presented with RFM from 24 weeks' gestation. The e-learning education package was created by colleagues in National Health Service (NHS) Education Scotland who had expertise in postgraduate clinician education. A link to the e-learning package was emailed to all clinicians in the participating unit about 1 month before the intended implementation of the package. The management plan for identification and delivery of babies at high risk included cardiotocography (within 2 h of presentation), measurement of liquor volume (within 12 h of presentation), and a growth scan to estimate fetal weight and abdominal circumference on the next working day (unless this latter had been done within the preceding 3 weeks). We encouraged maternity units to use umbilical artery Doppler in addition to the growth scan if such facilities were available. Delivery (with senior clinician input into decision making) was recommended for women who were at or after 37 weeks' gestation with any of estimated fetal weight below the 10th centile, abdominal circumference below the 10th centile, a liquor volume in which the deepest pool was less than 2 cm, abnormal carditocograph, or recurrent RFM. Management of other scenarios was as indicated in the appendix and in the protocol,^17^ with senior clinician input for women at less than 37 weeks' gestation who had additional risk factors on investigation.

Outcome data, potential confounders, and effect modifiers were derived from routinely collected hospital data (database codes are described in the appendix). Data were anonymised at the source and transferred at yearly intervals by secure file transfer to a dedicated AFFIRM project area in the NHS Scotland electronic Data Research and Innovation Service (eDRIS),^18^ where statistical analysis was done at the end of the study. We used the Intergrowth international standards^19^ to define growth centiles. Additional detail, reported to comply with the RECORD statement, is supplied in the appendix.^20^ No attempt was made to assess safety or adverse events in real time.

### Outcomes

The primary outcome measure was the incidence of stillbirth (babies delivered without signs of life after less than 24 weeks' gestation, or, if gestation was unknown, weighing 500 g or more). Pre-specified secondary outcomes were stillbirth at 37 weeks' gestation and above; stillbirth at 28 weeks' gestation and above (WHO definition of stillbirth); stillbirth at 22 weeks' gestation and above (international stillbirth alliance definition); stillbirths among healthily formed infants of 22 weeks' gestation and above, 24 weeks' gestation and above, 28 weeks' gestation and above, and 37 weeks' gestation and above; perinatal mortality (defined as stillbirth at 24 weeks' gestation and above and deaths in the first 7 days of life); number of caesarean sections; induction of labour (for any indication); number of elective deliveries (induction of labour and caesarean section before the onset of labour) overall; induction of labour at 39 weeks' gestation or later; mean gestation at induction of labour; number of admissions to the neonatal unit (and their reasons); number of admissions to the neonatal unit for more than 48 h; number of admissions to the neonatal unit for term babies (those born at 37 weeks 0 days or greater); proportion of infants with birthweight less than the tenth centile, customised for sex, remaining undelivered at or after 40 weeks' gestation; birthweight centile (according to the Intergrowth birthweight centile calculator); and number of spontaneous vaginal deliveries. Other secondary outcomes were the baby parameters: gestation at birth; proportion of babies born preterm (<37 weeks' gestation); sex of the baby; birthweight of the baby; Apgar score at 5 min; proportion of babies with 5 min Apgar score less than 7; proportion of babies with 5 min Apgar score less than 4; and resuscitation required at birth.^17^

### Statistical analysis

Our pre-planned sample size was sufficient to show a reduction in the incidence of stillbirth of at least 25%, as described in the appendix and in the protocol.^17^ In practice, we anticipated a stillbirth risk reduction of 30% on the basis of findings by Tveit and colleagues^13^ in Norway.

The primary analysis was done according to the intention-to-treat principle, with births analysed according to whether they took place during the control or intervention periods, irrespective of whether the intervention had been implemented as planned. Secondary on-treatment analyses assigned a birth to the control period if a site was non-adherent to the AFFIRM intervention at the time of the birth.

Stillbirth outcomes were summarised as the number of stillbirths per 1000 livebirths. Binary outcomes were analysed by generalised linear mixed model logistic regression to estimate the adjusted OR (aOR) and 95% CI for the intervention period versus the control period. A random effect was included for cluster, and the intervention and study time periods were fixed effects. We also adjusted for maternal age and multifetal pregnancies as potential confounders. Absolute and relative risk differences were calculated to help interpret the results. Continuous outcomes were analysed using a normal linear mixed model with the same structure, the intervention effect being expressed as the adjusted mean difference and 95% CI. The full statistical analysis plan is described in the appendix. There was no planned imputation of missing values, with the exception of smoking status during pregnancy. When missing, this information was imputed using smoking status in early pregnancy.

Adherence to the intervention (a potential effect modifier) was categorised as a binary variable on the basis of results of a questionnaire sent to the lead investigator. We asked whether the site had implemented the e-learning education package for staff, issued RFM leaflets to pregnant women, and implemented any of the other three specific aspects of the management plan in line with the protocol. Sites that largely implemented at least four of five of these aspects of the AFFIRM intervention were categorised as adherent, and those sites that had implemented less than four of the aspects were categorised as non-adherent. We also asked about the timing of implementing the intervention. In an on-treatment analysis, we considered (simultaneously) insufficient implementation of the intervention throughout the trial as well as sufficient implementation of the intervention but not at the assigned time.

We used SAS version 9.4 for all statistical analyses. This trial is registered with www.ClinicalTrials.gov, number NCT01777022.

### Role of the funding source

The funders of the study had no role in study design, data collection, data analysis, data interpretation, or writing of the report. The corresponding author had full access to all the data in the study and had final responsibility for the decision to submit for publication.

## Results

37 maternity hospitals were enrolled in the study (figure). Four hospitals withdrew before the study control period and provided no data. The reasons for withdrawal were lack of staff (largely lack of sonographer time, but also concerns about midwifery and obstetrician time) and cost implications (again, largely around perceived additional ultrasound scanning costs). 33 maternity hospitals were randomised to an implementation date. Data from 409 175 women delivering in the remaining 33 hospitals between Jan 1, 2014, and Dec 31, 2016, were included in the primary intention-to-treat analysis. No woman asked for her data to be excluded, and there were no other deviations from the protocol other than poor compliance. 87 of 409 175 datapoints (0·02%) were missing for the primary outcome analysis.

**Figure:**
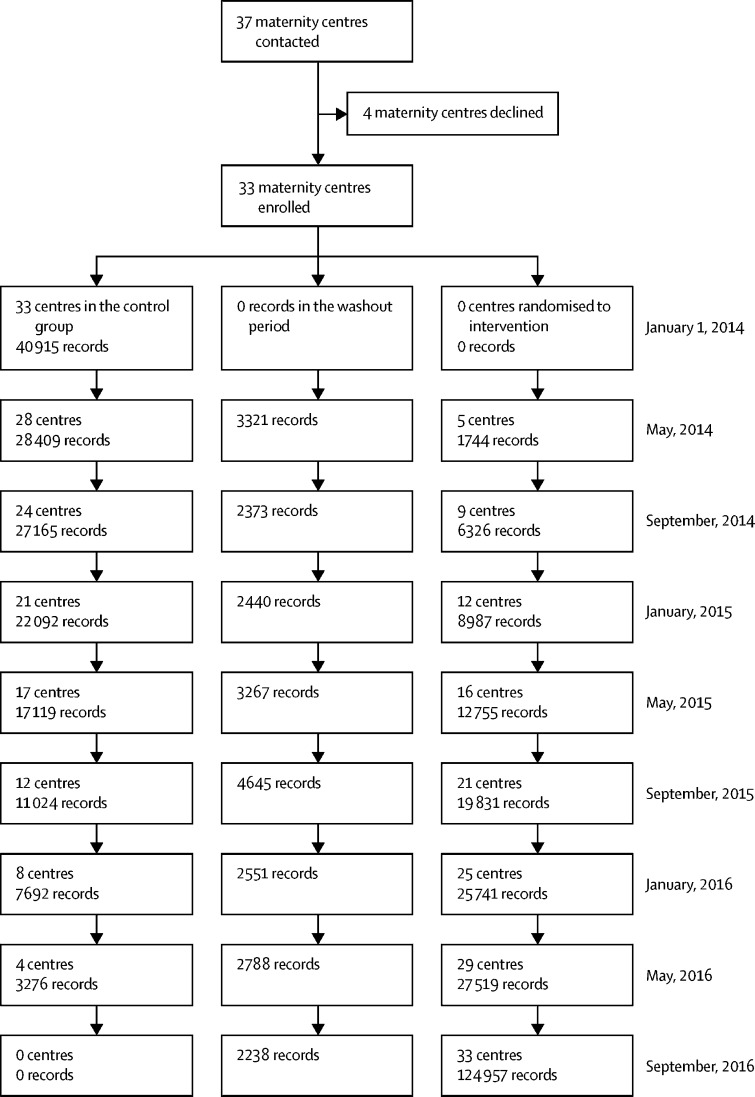
Trial profile

409 175 women delivered during the study (157 692 deliveries during the control period, 23 623 deliveries in the washout period, and 227 860 deliveries in the intervention period). There were no obvious differences in characteristics between the intervention groups (table 1).

**Table 1:** Population characteristics and mother and baby secondary endpoints by intervention period

		**Intervention (n=227 860)**	**Control (n=157 692)**	**Washout (n=23 623)**	**Overall (n=409 175)**
Maternal age, years		30·2 (5·7)	30·0 (5·8)	30·0 (5·8)	30·1 (5·8)
Ethnicity					
	White	169 531 (76·9%)	118 127 (76·6%)	16 998 (73·8%)	304 656 (76·6%)
	Mixed	3221 (1·5%)	2845 (1·8%)	378 (1·6%)	6444 (1·6%)
	Asian	15 144 (6·9%)	10 966 (7·1%)	1670 (7·2%)	27 780 (7·0%)
	Black African	3612 (1·6%)	3019 (2·0%)	386 (1·7%)	7017 (1·8%)
	Black Caribbean	2560 (1·2%)	1269 (0·8%)	322 (1·4%)	4151 (1·0%)
	Arab or other ethnic group	4126 (1·9%)	2272 (1·5%)	493 (2·1%)	6891 (1·7%)
Body-mass index					
	Underweight (<18·5 kg/m^2^)	5107 (2·7%)	3605 (2·8%)	526 (2·6%)	9238 (2·7%)
	Normal (≥18·5 to 24·9 kg/m^2^)	90 266 (47·3%)	63 055 (49·0%)	9639 (48·6%)	162 960 (48·0%)
	Overweight (≥25 to 29·9 kg/m^2^)	53 829 (28·2%)	35 876 (27·9%)	5605 (28·2%)	95 310 (28·1%)
	Obese (≥30 kg/m^2^)	41 584 (21·8%)	26 074 (20·3%)	4082 (20·6%)	71 740 (21·1%)
Smoking during pregnancy		28 620 (13·7%)	21 509 (14·3%)	3182 (14·9%)	53 311 (14·0%)
Parity					
	0	89 822 (40·8%)	65 145 (42·4%)	9208 (40·2%)	164 175 (41·4%)
	≥1	130 414 (59·2%)	88 423 (57·6%)	13 711 (59·8%)	232 548 (58·6%)
Decile of deprivation (Scottish Index of Multiple Deprivation 2016)					
	1 most deprived	13 565 (14·0%)	6686 (12·9%)	1215 (13·6%)	21 466 (13·6%)
	2	11 828 (12·2%)	6338 (12·2%)	1103 (12·4%)	19 269 (12·2%)
	3	10 719 (11·1%)	5676 (10·9%)	1022 (11·5%)	17 417 (11·1%)
	4	9710 (10·0%)	5348 (10·3%)	853 (9·6%)	15 911 (10·1%)
	5	8751 (9·0%)	4975 (9·6%)	783 (8·8%)	14 509 (9·2%)
	6	8212 (8·5%)	4587 (8·8%)	816 (9·2%)	13 615 (8·6%)
	7	8407 (8·7%)	4847 (9·3%)	792 (8·9%)	14 046 (8·9%)
	8	8449 (8·7%)	4733 (9·1%)	800 (9·0%)	13 982 (8·9%)
	9	8612 (8·9%)	4687 (9·0%)	773 (8·7%)	14 072 (8·9%)
	10 (least deprived)	8490 (8·8%)	3985 (7·7%)	754 (8·5%)	13 229 (8·4%)
Estimated gestation, weeks		39·0 (2·2)	39·1 (2·2)	39·0 (2·3)	39·0 (2·2)
Estimated gestation					
	≤24 weeks	668 (0·3%)	387 (0·3%)	65 (0·3%)	1120 (0·3%)
	>24 to ≤28 weeks	1164 (0·5%)	734 (0·5%)	124 (0·5%)	2022 (0·5%)
	>28 to ≤32 weeks	2416 (1·1%)	1570 (1·0%)	272 (1·2%)	4258 (1·1%)
	>32 to ≤34 weeks	3341 (1·5%)	2158 (1·4%)	344 (1·5%)	5843 (1·4%)
	>34 to ≤37 weeks	26 203 (11·5%)	16 117 (10·4%)	2604 (11·1%)	44 924 (11·1%)
	>37 to ≤39 weeks	90 767 (40·0%)	59 354 (38·5%)	9262 (39·3%)	159 383 (39·4%)
	>39 to ≤41 weeks	97 753 (43·1%)	70 249 (45·5%)	10 384 (44·1%)	178 386 (44·1%)
	>41 weeks	4658 (2·1%)	3687 (2·4%)	502 (2·1%)	8847 (2·2%)
Number of births this pregnancy					
	1	224 066 (98·3%)	155 117 (98·4%)	23 244 (98·4%)	402 427 (98·4%)
	>1	3794 (1·7%)	2575 (1·6%)	379 (1·6%)	6748 (1·6%)
Mode of delivery					
	Spontaneous vaginal delivery	130 658 (57·4%)	94 337 (59·8%)	13 733 (58·1%)	238 728 (58·4%)
	Assisted vaginal delivery	28 171 (12·4%)	18 413 (11·7%)	2932 (12·4%)	49 516 (12·1%)
	Elective caesarean section	30 576 (13·4%)	18 366 (11·6%)	2975 (12·6%)	51 917 (12·7%)
	Emergency caesarean section	33 996 (14·9%)	21 865 (13·9%)	3412 (14·4%)	59 273 (14·5%)
	Other or unspecified	4402 (1·9%)	4673 (3·0%)	569 (2·4%)	9644 (2·4%)
Induction of labour					
	None	116 312 (58·2%)	87 160 (63·6%)	12 403 (59·5%)	215 875 (60·3%)
	ARM or ARM and OXY	10 069 (5·0%)	5822 (4·2%)	1066 (5·1%)	16 957 (4·7%)
	Oxytocics	2798 (1·4%)	1747 (1·3%)	325 (1·6%)	4870 (1·4%)
	Any prostaglandin	36 453 (18·2%)	17 500 (12·8%)	3202 (15·3%)	57 155 (16·0%)
	Other or unknown	34 179 (17·1%)	24 883 (18·1%)	3865 (18·5%)	62 927 (17·6%)
Any fetal abnormality		10 653 (5·1%)	5319 (4·0%)	898 (4·0%)	16 870 (4·6%)
Sex of baby					
	Male	118 579 (51·2%)	81 769 (51·0%)	12 426 (51·8%)	212 774 (51·1%)
	Female	112 932 (48·7%)	78 465 (48·9%)	11 565 (48·2%)	202 962 (48·8%)
	Not determined	209 (0·1%)	87 (0·1%)	20 (0·1%)	316 (0·1%)
Birthweight categories					
	≤2500 g	18 159 (7·9%)	12 310 (7·7%)	1878 (7·8%)	32 347 (7·8%)
	>2500 g to <3500 g	112 915 (48·9%)	79 150 (49·5%)	11 806 (49·3%)	203 871 (49·1%)
	>3500 g to <4000 g	71 295 (30·9%)	48 674 (30·4%)	7245 (30·3%)	127 214 (30·7%)
	≥4000 g	28 704 (12·4%)	19 787 (12·4%)	3020 (12·6%)	51 511 (12·4%)
Birthweight, g		3353·2 (626·5)	3351·4 (621·2)	3348·6 (632·5)	3352·2 (624·8)
Birthweight centiles					
	≤10%	10 853 (4·7%)	8444 (5·4%)	1216 (5·1%)	20 513 (5·0%)
	>10% to 90%	168 425 (73·4%)	114 645 (73·6%)	17 483 (73·5%)	300 553 (73·5%)
	≥90%	50 178 (21·9%)	32 770 (21·0%)	5095 (21·4%)	88 043 (21·5%)
Apgar score at 5 min					
	0	241 (0·1%)	168 (0·1%)	29 (0·1%)	438 (0·1%)
	1	184 (0·1%)	136 (0·1%)	20 (0·1%)	340 (0·1%)
	2	187 (0·1%)	110 (0·1%)	19 (0·1%)	316 (0·1%)
	3	255 (0·1%)	138 (0·1%)	22 (0·1%)	415 (0·1%)
	4	397 (0·2%)	233 (0·2%)	28 (0·1%)	658 (0·2%)
	5	783 (0·4%)	505 (0·3%)	90 (0·4%)	1378 (0·3%)
	6	1566 (0·7%)	1071 (0·7%)	169 (0·7%)	2806 (0·7%)
	7	2529 (1·2%)	1829 (1·2%)	279 (1·2%)	4637 (1·2%)
	8	5167 (2·4%)	3666 (2·4%)	575 (2·5%)	9408 (2·4%)
	9	131 479 (60·0%)	80 802 (52·7%)	12 685 (55·5%)	224 966 (56·9%)
	10	76 464 (34·9%)	64 748 (42·2%)	8948 (39·1%)	150 160 (38·0%)
Apgar at 5 min <4		867 (0·4%)	552 (0·4%)	90 (0·4%)	1509 (0·4%)
Apgar at 5 min <7		3613 (1·6%)	2361 (1·5%)	377 (1·6%)	6351 (1·6%)
Resuscitation used		13 589 (7·6%)	8435 (6·9%)	1372 (7·4%)	23 396 (7·3%)

Data are n (%) or mean (SD). Denominator for characteristics up to and including fetal abnormality is number of mothers. From sex of baby and onwards, the denominator is number of babies. For baby data, N=160 465 in the control group, N=231 813 in the intervention group, N=24 022 in the washout group, and N=416 300 overall. Data are missing for maternal age (916 [0·2%]), ethnicity (52 236 [12·8%]), body-mass index (69 927 [17·1%]), smoking during pregnancy (28 592 [7·0%]), parity 12 452 [3·0%]), decile of deprivation (378 of 157 894 Scotland participants [0·2%]), estimated gestation (4373 [1·1%]), mode of delivery (97 [<0·1%]), induction of labour (51 391 [12·6%]), any fetal abnormality (44 269 [10·8%]), sex of baby (248 [0·1%]), birthweight (1357 [0·3%]), birthweight centile (7191 [1·7%]), Apgar at 5 min (20 778 [5·0%]), and resuscitation used (96 385 [23·2%]). The Scottish Index of Multiple Deprivation 2016 is shown for participants in Scotland only and ranks small geographical areas from most deprived (ranked 1) to least deprived (ranked 6976). ARM=artificial rupture of membranes. OXY=oxytocin.

The incidence of stillbirth at or beyond 24 weeks was 4·06 per 1000 births during the intervention period and 4·40 per 1000 births during the control period (aOR 0·90, 95% CI 0·75–1·07; p=0·232; table 2). The intervention had no significant effect on the incidence of stillbirths when using alternative gestational age thresholds for stillbirth or restricting stillbirths to normally formed infants, and we found no effect on perinatal mortality (table 2). Induction of labour and caesarean section (secondary outcomes) were more common during the intervention period than during the control period (table 3). Admissions to the neonatal unit were similar during both periods, although more babies were admitted for more than 48 h during the intervention period than during the control period (table 3).

**Table 2:** Stillbirth and perinatal mortality

		**Intervention (n=227 860)**	**Control (n=157 692)**	**Adjusted OR (95% CI)**	**p value**	**Absolute effect (95% CI) per 10 000 pregnancies**
Livebirths		226 895	156 963	..	..	..
Stillbirths at ≥24 weeks' gestation, n (per 1000 livebirths; primary outcome*)		921 (4·06)	691 (4·40)	0·90 (0·75–1·07)	0·232	5 fewer (11 fewer to 3 more)
Stillbirths, n (per 1000 livebirths; secondary outcome)						
	≥22 weeks' gestation*	933 (4·11)	704 (4·49)	0·89 (0·75–1·07)	0·213	5 fewer (11 fewer to 3 more)
	≥28 weeks' gestation	679 (2·99)	512 (3·26)	0·97 (0·79–1·18)	0·759	1 fewer (7 fewer to 6 more)
	≥37 weeks' gestation	281 (1·24)	229 (1·46)	0·94 (0·69–1·26)	0·662	1 fewer (4 fewer to 4 more)
	≥22 weeks' gestation in normally formed infants*	779 (3·43)	537 (3·42)	0·98 (0·80–1·21)	0·855	1 fewer (7 fewer to 7 more)
	≥24 weeks' gestation in normally formed infants*	771 (3·40)	528 (3·36)	0·98 (0·79–1·21)	0·825	1 fewer (7 fewer to 7 more)
	≥28 weeks' gestation in normally formed infants	570 (2·51)	404 (2·57)	1·02 (0·80–1·29)	0·893	0 fewer (5 fewer to 7 more)
	≥37 weeks' gestation in normally formed infants	239 (1·05)	189 (1·20)	0·88 (0·61–1·24)	0·457	2 fewer (5 fewer to 3 more)
Perinatal mortality, n (per 1000 births)		1238 (6·21)	923 (6·82)	0·98 (0·83–1·17)	0·861	1 fewer (12 fewer to 12 more)

ORs are presented for intervention versus control such that an OR less than 1 indicates a benefit for the intervention on the stillbirth outcomes. ORs are adjusted for maternal age, number of babies in the pregnancy, and study time period and cluster. All livebirths were included in the denominator, irrespective of estimated gestation or weight. Data are missing for stillbirth ≥24 weeks' gestation (82 [0·02%]), stillbirth ≥22 weeks' gestation (57 [0·01%]), stillbirth ≥ 28 weeks gestation (503 [0·13%]); stillbirth ≥37 weeks' gestation (1184 [0·31%]); stillbirth ≥22 weeks' gestation in normally formed infants (378 [0·10%]), stillbirth ≥24 weeks' gestation in normally formed infants 395 [0·10%]), stillbirth ≥28 weeks' gestation in normally formed infants 720 [0·19%]), stillbirth ≥37 weeks' gestation in normally formed infants (1266 [0·33%]); perinatal mortality 50 828 (13·2%]).

* If estimated gestation was missing, babies weighing 500 g or more at delivery were included in the numerator for stillbirths at 22 weeks' or 24 weeks' gestation or more, but not for stillbirths at 28 weeks' or 37 weeks' gestation. OR=odds ratio.

**Table 3:** Pregnancy and baby secondary outcomes

	**Intervention (n=227 860)**	**Control (n=157 692)**	**Adjusted OR (95% CI)**	**p value**	Absolute effect (95% CI) per 10 000 pregnancies or per 10 000 babies*
Preterm pregnancy	17 376 (7·7%)	11 228 (7·3%)	1·05 (1·00–1·10)	0·050	34 more (0–68 more)
Caesarean section	64 572 (28·3%)	40 231 (25·5%)	1·09 (1·06–1·12)	<0·0001	162 more (105–218 more)
Induction at ≥39 weeks' gestation	57 815 (39·8%)	33 317 (33·6%)	1·08 (1·04–1·11)	<0·0001	165 more (88–245 more)
Induction of labour	83 499 (40·7%)	49 952 (35·8%)	1·05 (1·02,1·08)	0·0015	108 more (41–177 more)
Elective delivery	111 837 (54·6%)	67 227 (48·2%)	1·04 (1·01–1·07)	0·0123	91 more (20–160 more)
Elective delivery at ≥39 weeks' gestation	76 247 (52·4%)	44 838 (45·2%)	1·05 (1·02–1·09)	0·0022	128 more (47–212 more)
Spontaneous vaginal delivery	130 658 (57·4%)	94 337 (59·8%)	0·90 (0·88–0·92)	<0·0001	256 fewer (319–194 fewer)
Admitted to neonatal unit	19 237 (10·1%)	13 029 (10·1%)	1·02 (0·97–1·07)	0·504	14 more (28 fewer to 59 more)*
Admitted to neonatal unit for >48 h	12 676 (6·7%)	8041 (6·2%)	1·12 (1·06–1·18)	0·0001	68 more (32 to 105 more)*
Admitted to neonatal unit at ≥37 weeks' gestation	10 384 (6·0%)	7497 (6.5%)	0·95 (0·89–1·01)	0·091	32 fewer (66 fewer to 5 more)*
Small for gestational age (≤10th centile) delivered ≥40 weeks' gestation	3461 (1·5%)	3081 (2·0%)	0·86 (0·78–0·95)	0·0009	27 fewer (42–10 fewer)*
Preterm baby	19 815 (8·6%)	12 738 (8·1%)	1·05 (1·00–1·10)	0·061	34 more (1 fewer to 72 more)*

Data are n (%). ORs are adjusted for maternal age, number of babies in the pregnancy and study time period and cluster. Data are missing for preterm pregnancy (4307 [1·1%]), caesarean section (95 [0·02%]), induction at ≥39 weeks (140 930 [36·6%]), induction of labour (41 183 [10·7%]); elective delivery (41 239 [10·7%]), elective delivery at ≥39 weeks' gestation 140 945 [36·6%]), spontaneous vaginal delivery (95 [0·02%]), admitted to neonatal unit (72 405 [18·5%]), admitted to neonatal unit for >48h (72 405 [18·5%]), admitted to neonatal unit at ≥37 weeks' gestation (103 029 [26·3%]), small for gestational age (≤10th centile) delivered ≥40 weeks' gestation (6963 [1·8%]), and preterm baby 4372 [1·1%]). OR=odds ratio.

* Absolute effect sizes are per 10 000 babies for outcomes of neonatal unit admission, born small for gestational age, or preterm baby.

The incidence of preterm births and number of preterm babies were similar during both periods, but the mean gestational age of birth was slightly lower in the intervention group (adjusted mean difference −0·35 days, 95% CI −0·53 to −0·16; table 4). 3081 of 157 692 (2·0%) babies born during the control period and 3461 of 227 860 babies (1·5%) born during the intervention period were small for gestational age at birth but not delivered until or after 40 weeks' gestation (p=0·001). Relative risks are reported in the appendix.

**Table 4:** Adjusted mean differences in estimated gestation and birthweight centiles

	**Intervention**	**Control**	**Adjusted mean difference (95% CI)**	**p value**
Estimated gestation, weeks	39·0 (2·2)	39·1 (2·2)	−0·05 (−0·08 to −0·02)	0·0003
Estimated gestation for inductions only, weeks	39·1 (2·2)	39·3 (2·0)	−0·03 (−0·07 to 0·02)	0·260
Birthweight centile	63·4 (28·1)	62·1 (28·6)	0·56 (0·21 to 0·90)	0·002

Mean differences (intervention–control) were calculated after adjusting for maternal age, number of babies in the pregnancy, study time periods, and cluster. Data are missing for estimated gestation (4307 [1·1%]), estimated gestation for inductions only (3566 [2·7%]), and for birthweight centile 6735 [1·7%]).

The results of the on-treatment analysis were similar to the main intention-to-treat analysis. 550 of 140 888 births during the intervention period ended in stillbirths (3·90 stillbirths per 1000 livebirths). During the control period, 1084 of 251 251 births at or beyond 24 weeks' gestation were stillbirths (4·31 stillbirths per 1000 livebirths), giving aOR 0·88 (95% CI 0·76–1·02). In the on-treatment analysis, we found no significant difference between groups in the incidence of births of babies that were small for gestational age. During the intervention period, 2120 of 141 480 newborn babies (1·5%) were small for gestational age, compared with 4636 of 252 377 newborn babies (1·9%) during the control period (p=0·153; appendix). The effect of the intervention in the compliant and non-compliant groups is described in the appendix.

## Discussion

A package of interventions with strategies for increasing pregnant women's reporting when they perceive RFM, combined with a management plan to identify and minimise further risk, including early delivery where relevant, did not reduce the incidence of stillbirth at or beyond 24 weeks' gestation or perinatal mortality. The intervention increased the frequency of labour induction and birth by caesarean section and prolonged neonatal unit admission period.

In preparation for this study, we completed a literature review in which we found no other randomised trial that had assessed RFM as an alert of increased stillbirth in combination with a management plan to refine and or reduce the risk. The strengths of our study are that it was conducted with little evidence of bias and a low potential for unmeasured confounding. We adjusted for the potential confounders of maternal age, number of babies in the pregnancy, time (ie, month and year of birth), and (in Scotland only) deprivation. We were unable to adjust for other potential confounders (eg, other maternal characteristics). Importantly, very few data were missing in our primary outcome or in variables we used in adjustment. Misclassification (at least for the primary outcome) is likely to be negligible.^21^

AFFIRM was a population-based study of women delivering in regional hospitals. Eligibility did not change over time, and the study analysis adjusted for any secular trend in frequency of stillbirth, so we believe the risk of unmeasured confounding is small. Generalisability of the study (at least in the UK) is demonstrated by similar incidence of stillbirth in participating hospitals as that calculated for the UK as a whole by the MBRRACE UK investigators 4·73 stillbirths per 1000 livebirths in 2014; *vs* 4·56 per 1000 livebirths in 2015 *vs* 4·52 per 1000 livebirths in 2016).

This study has limitations. Although more than 400 000 women were included, a 30% reduction in the incidence of stillbirth was the smallest effect size we could expect to detect. Our sample size was informed by the expected effect size from previous data.^13^ Although the apparent trend toward a 10% relative risk reduction in stillbirth (five fewer stillbirths per 10 000 [range 11 fewer to three more]) might be considered to conceal a small true benefit, we detected no trend toward reduced perinatal mortality overall (OR 0·98), not even when the analysis was restricted to normally formed infants. Further limitations are that the intervention package might not have been sufficiently effective to initiate behaviour change in clinicians and in pregnant women. Adherence was imperfect, with 13 maternity centres (39·4%) adhering to four or fewer of the five components of the intervention. Our assessment of adherence is also subject to recall and reporting bias by local principal investigators.

This study will re-ignite the controversy about the efficacy of RFM awareness to reduce stillbirth and the underlying mechanisms linking RFM and stillbirth. With a population of more than 400 000 women, we showed that RFM awareness did not significantly reduce the risk of stillbirth. It is possible that the absence of a significant reduction in stillbirth risk is because RFM is a symptom of inevitable fetal death, irrespective of any subsequent action. If so, it will never be possible to use RFM as an alert to prompt action and improve outcome, and no strategy around RFM awareness will be effective in reducing the risk of stillbirth. We believe this possibility is small because of the reduction in the incidence of babies born small for gestational age (a group of babies at high risk for stillbirth) who were not born until or after 40 weeks' gestation in the intervention group. This reduction in babies born small for gestational age is consistent with the concept that AFFIRM correctly identified a group of high-risk babies with placental insufficiency, achieved timely delivery, and prevented stillbirth. About 23% of stillbirths in high-income countries are attributable to small size for gestational age (<10th centile).^22^ Our use of the Intergrowth international standards^19^ to define growth centiles led to 5% of babies overall being defined as below the 10th centile for gestational age. This proportion is similar to those found in other resource-rich countries such as New Zealand, Germany, and Sweden.^23^, ^24^

Several other similar trials are underway, including the My Baby's Movements trial in Australia and New Zealand, and the Mindfetalness study^25^ in Sweden, with an estimated combined sample size of 300 000. Meta-analysis of the data from these studies (either conventional or using individual patient data) will increase the power to detect (or exclude) a smaller effect size. Future research directions will best be informed by summary data from all these trials combined. In the meantime, we plan an economic analysis of the effect of the AFFIRM intervention.

Although we found no significant effect of the intervention on the risk of stillbirth, it did increase the frequency of birth by induced labour (at term and overall) and caesarean section, and it was associated with prolonged (>48 h) duration of admission to the neonatal unit. Mean gestation decreased, but the incidence of prematurity did not increase significantly. Again, it is possible that the finding of a significant effect on these process variables, but not on the outcome of stillbirth, is a power issue given the greater frequency of these process variables compared with the outcome of stillbirth. At present, because timely delivery is the only strategy to prevent stillbirth in response to concerns about fetal wellbeing, any package of care that relies on a test of fetal wellbeing to reduce stillbirth could increase the frequency of elective delivery, unless the false positive rate of the test is negligible. In other words, for most tests of fetal wellbeing, there might be a trade-off between stillbirths prevented and the increased number of elective deliveries initiated, although this was not the case in the Frøen study.^13^ The magnitude of this trade-off for using RFM as an alert (the effect on incidence of stillbirth and process variables in a population of 10 000 women) is shown in Table 2, Table 3.

The AFFIRM study is one of the few randomised trials with stillbirth as the primary outcome. Our findings show that a cluster design with routinely collected data is feasible. Our highly novel design used anonymised data at scale (resulting in a very efficient trial), which we believe provides a template for further trials. The cluster methodology allows interventions to be implemented at a hospital or regional level and minimises contamination between groups, which might occur if clinicians have to provide different standards of care to different women on the same day. The stepped wedge design had the benefit of allowing all maternity units to adopt (and persist with) the intervention and is ideal for interventions that are difficult to unlearn or to abandon. Randomising maternity units to the timing of uptake of intervention minimised bias but might have created additional opportunities for poor adherence, since compliance with the timing of adoption of the intervention as well as compliance with the intervention can both be suboptimal. Correct timing was difficult to achieve in AFFIRM; although sites were enthusiastic about participating at the beginning of the study, the practicalities (and costs) of implementing the intervention sometimes delayed the onset. Adherence to the ultrasound components was the most difficult aspect of compliance with the intervention, largely because of shortage of ultrasonography staff. We do not believe this had a major effect on the results, given the similarities in results between the intention-to-treat and on-treatment analyses.

A further potential limitation is uncertainty about whether the washout period was long enough to facilitate implementation. The AFFIRM findings also show the challenges of any study to address stillbirth. The relatively low risk of stillbirth but high incidence of other outcomes, such as prolonged neonatal unit admission and caesarean section, can result in higher power to show harms (such as increased intervention) rather than benefit.

The data on the effect of our RFM package on risk of stillbirth, caesarean section, induction of labour, and neonatal unit admission in a notional population of 10 000 women will be of interest to pregnant women, clinicians, policy makers, and commissioning groups. Further research to identify better predictive tests for stillbirth (to enable targeting of the only current treatment of earlier delivery) is urgently needed.

The intervention package, in its present form, was not effective; it led to a significant increase in interventions and cannot be recommended. Other studies on the efficacy of RFM strategies are ongoing and, together with the AFFIRM findings, will provide the best evidence on the likely effectiveness of RFM awareness as a stillbirth reduction strategy and can help clinicians and policy makers make informed decisions as to how RFM awareness might fit into a stillbirth reduction strategy.

## References

[1] de Bernis L, Kinney MV, Stones W (2016). Stillbirths: ending preventable deaths by 2030. Lancet.

[2] Flenady V, Middleton P, Smith GC (2011). Stillbirths: the way forward in high-income countries. Lancet.

[3] Warland J, O'Brien LM, Heazell AE, Mitchell EA, for the Stillbirth Consortium (2015). An international internet survey of the experiences of 1,714 mothers with a late stillbirth: the STARS cohort study. BMC Pregnancy Childbirth.

[4] Efkarpidis S, Alexopoulos E, Kean L (2004). Case-control study of factors associated with intrauterine fetal deaths. MedGenMed.

[5] Dutton P, Warrander LK, Roberts S (2012). Predictors of poor perinatal outcome following maternal perception of reduced fetal movement—a prospective cohort study. PLoS One.

[6] Warrander LK, Batra G, Bernatavicius G (2012). Maternal perception of reduced fetal movements is associated with altered placental structure and function. PLoS One.

[7] Winje BA, Roald B, Kristensen NP, Froen JF (2012). Placental pathology in pregnancies with maternally perceived decreased fetal movement— a population-based nested case-cohort study. PLoS One.

[8] Stacey T, Thompson JM, Mitchell EA (2011). Maternal perception of fetal activity and late stillbirth risk: findings from the Auckland Stillbirth Study. Birth.

[9] Heazell AEP, Warland J, Stacey T (2017). Stillbirth is associated with perceived alterations in fetal activity—findings from an international case control study. BMC Pregnancy Childbirth.

[10] Grant A, Elbourne D, Valentin L, Alexander S (1989). Routine formal fetal movement counting and risk of antepartum late death in normally formed singletons. Lancet.

[11] Mangesi L, Hofmeyr GJ, Smith V, Smyth RM (2015). Fetal movement counting for assessment of fetal wellbeing. Cochrane Database Syst Rev.

[12] Heazell AE, Froen JF (2008). Methods of fetal movement counting and the detection of fetal compromise. J Obstet Gynaecol.

[13] Tveit JV, Saastad E, Stray-Pedersen B (2009). Reduction of late stillbirth with the introduction of fetal movement information and guidelines—a clinical quality improvement. BMC Pregnancy Childbirth.

[14] Heazell AE, Whitworth MK, Whitcombe J (2015). Research priorities for stillbirth: process overview and results from UK Stillbirth Priority Setting Partnership. Ultrasound Obstet Gynecol.

[15] Heazell AE, Green M, Wright C (2008). Midwives' and obstetricians' knowledge and management of women presenting with decreased fetal movements. Acta Obstet Gynecol Scand.

[16] Draper E, Kurinczuk JJ, Kenyon S, on behalf of MBRRACE-UK (2017). MBRRACE-UK 2017 perinatal confidential enquiry: term, singleton, intrapartum stillbirth and intrapartum-related neonatal death.

[17] Heazell AEP, Weir CJ, Stock SJE (2017). Can promoting awareness of fetal movements and focusing interventions reduce fetal mortality? A stepped-wedge cluster randomised trial (AFFIRM). BMJ Open.

[18] NHS Scotland Information Services Division (2017). Use of the NSS National Safe Haven. http://www.isdscotland.org/Products-and-Services/EDRIS/Use-of-the-National-Safe-Haven/.

[19] Villar J, Cheikh Ismail L, Victora CG (2014). International F, Newborn Growth Consortium for the 21st C. International standards for newborn weight, length, and head circumference by gestational age and sex: the Newborn Cross-Sectional Study of the INTERGROWTH-21st Project. Lancet.

[20] Benchimol EI, Smeeth L, Guttmann A (2015). The reporting of studies conducted using observational routinely-collected health data (RECORD) statement. PLoS Med.

[21] NHS Scotland Information Services Division 2010 Data Quality Assurance, Assessment of Maternity Data, (SMR02), 2008–09. http://www.isdscotland.org/Products-and-Services/Data-Quality/Previous-Projects/DQA-Assessment-of-Maternity-Data-SMR02-2008-to-2009.pdf.

[22] Flenady V, Koopmans L, Middleton P (2011). Major risk factors for stillbirth in high-income countries: a systematic review and meta-analysis. Lancet.

[23] Anderson NH, Sadler LC, McKinlay CJD, McCowan LME (2016). INTERGROWTH-21st vs customized birthweight standards for identification of perinatal mortality and morbidity. Am J Obstet Gynecol.

[24] Francis A, Hugh O, Gardosi J (2018). Customized vs INTERGROWTH-21(st) standards for the assessment of birthweight and stillbirth risk at term. Am J Obstet Gynecol.

[25] Radestad I, Akselsson A, Georgsson S (2016). Rationale, study protocol and the cluster randomization process in a controlled trial including 40,000 women investigating the effects of mindfetalness. Sex Reprod Healthc.

