# Department of Error

**DOI:** 10.1016/S0140-6736(20)31973-5

**Published:** 2020-10-24

*Norman JE, Heazell AEP, Rodriguez A, et al. Awareness of fetal movements and care package to reduce fetal mortality (AFFIRM): a stepped wedge, cluster-randomised trial.* Lancet *2018; **392:** 1629–38—*In table 2 of this Article, some data in the “Perinatal mortality, n (per 1000 births)” row have been amended in line with the definition for perinatal mortality given in the Outcomes section of the Methods, amends have been made to the legend to clarify the definition of stillbirths at ≥22 weeks' gestation, and the appendix has been corrected. These data changes did not affect the reported primary outcome data or change the overall summary or conclusions of the paper. These corrections have been made to the online version as of Oct 22, 2020.

