# Supplementary material for: Awareness of fetal movements and care package to reduce fetal mortality (AFFIRM): a stepped wedge, cluster-randomised trial
Source: Lancet. 2018 Nov 3;392(10158):1629–38. doi: 10.1016/S0140-6736(18)31543-5 (PMC6215771; doi:10.1016/S0140-6736(18)31543-5)
Supplement: Supplementary appendix [file mmc1.pdf]

THE LANCET

Supplementary appendix

This appendix formed part of the original submission and has been peer reviewed. We post it as supplied by the authors.

Supplement to: Norman JE, Heazell AEP, Rodriguez A, et al. Awareness of fetal movements and care package to reduce fetal mortality (AFFIRM): a stepped wedge, cluster-randomised trial. *Lancet* 2018; published online Sept 27. http://dx.doi.org/10.1016/S0140-6736(18)31543-5.

Supplementary Table 1.
Location, groupings and number of pregnancies in participating hospitals over the three year study period.

Study centre	Group	Intervention N=227860	Control N=157692	Washout N=23623	Overall N=409175
England 001	2	6771 (2·97)	1771 (1·12)	552 (2·34)	9094(2·22)
England 003	4	8063 (3·54)	3752 (2·38)	743 (3·15)	12558 (3·07)
England 004	2	6263 (2·75)	1676 (1·06)	541 (2·29)	8480 (2·07)
England 005	5	14006 (6·15)	11613 (7·36)	1567 (6·63)	27186 (6·64)
England 006	5	5127(2·25)	4359 (2·76)	568 (2·40)	10054 (2·46)
England 007	5	14975 (6·57)	6503 (4·12)	1732 (7·33)	23210 (5·67)
England 008	4	9055 (3·97)	5436 (3·45)	760 (3·22)	15251 (3·73)
England 009	7	7930 (3·48)	14321 (9·08)	1410 (5·97)	23661 (5·78)
England 011	8	2058 (0·90)	5823 (3·69)	479 (2·03)	8360 (2·04)
England 012	8	1686 (0·74)	4939 (3·13)	390 (1·65)	7015 (1·71)
Ireland 0011	1	14776 (6·48)	2053 (1·30)	994 (4·21)	17823 (4·36)
Ireland 0012	6	9802 (4·30)	12782 (8·11)	1412 (5·98)	23996 (5·86)
Ireland 0043	4	15842 (6·95)	9597 (6·09)	1426 (6·04)	26865 (6·57)
Scotland 001	5	5139 (2·26)	4565 (2·89)	571 (2·42)	10275 (2·51)
Scotland 002	1	2535 (1·11)	374 (0·24)	196 (0·83)	3105 (0·76)
Scotland 003	5	1831 (0·80)	1575 (1·00)	207 (0·88)	3613 (0·88)
Scotland 004	1	8486 (3·72)	1118 (0·71)	567 (2·40)	10171 (2·49)
Scotland 005	2	6902 (3·03)	1813 (1·15)	580 (2·46)	9295 (2·27)
Scotland 006	3	6927 (3·04)	2896 (1·84)	561 (2·37)	10384 (2·54)
Scotland 007	3	10806 (4·74)	5009 (3·18)	939 (3·97)	16754 (4·09)
Scotland 008	3	11478 (5·04)	4813 (3·05)	940 (3·98)	17231 (4·21)
Scotland 009	7	5036 (2·21)	9351 (5·93)	831 (3·52)	15218 (3·72)
Scotland 010	7	1009 (0·44)	1876 (1·19)	196 (0·83)	3081 (0·75)
Scotland 011	7	2026 (0·89)	3667 (2·33)	351 (1·49)	6044 (1·48)
Scotland 012	8	3307 (1·45)	9482 (6·01)	735 (3·11)	13524 (3·31)
Scotland 013	1	6705 (2·94)	881 (0·56)	465 (1·97)	8051 (1·97)
Scotland 014	1	16469 (7·23)	2225 (1·41)	1099 (4·65)	19793 (4·84)
Scotland 015	2	8366 (3·67)	2289(1.45)	700 (2·96)	11355 (2·78)
Wales 003	4	3611 (1·58)	1663 (1·05)	338 (1·43)	5612 (1·37)
Wales 004	8	2870 (1·26)	9008 (5·71)	634 (2·68)	12512 (3·06)
Wales 005	6	2423 (1·06)	3411 (2·16)	348 (1·47)	6182 (1·51)

Wales 006	6	3059 (1·34)	4005 (2·54)	442 (1·87)	7506 (1·83)
Wales 007	6	2521 (1·11)	3046 (1·93)	349 (1·48)	5916 (1·45)

Supplementary Table 2. Codes for congenital anomaly outcome data from ISD (Information Services Division, NHS Scotland)

ISD Group	NEW ISD Congenital anomaly groups	UPDATED GROUP NAME	ICD-9 Codes	ICD-10 Codes
1	Nervous System	Central Nervous System	740-742; (330*; 335*; 343*; 359* used if recorded on death certificate)	Q00-Q07
2	***** Neural Tube Defects	Neural Tube Defects	740, 741, 742·0	Q00, Q01, Q05
3	***** Anencephalus	Anencephalus	740	Q00
4	***** Spina bifida +/- Hydrocephalus	All Spina Bifida	741	Q05
5	***** Encephalocele	Encephalocele	742·0	Q01
6	*****Hydrocephalus	Hydrocephalus	742·3	Q03
7	Anomalies of the eye	Anomalies of the eye	743	Q10·0, Q10·4, Q10·6-Q10·7, Q11-Q15
8	Anomalies of the ear		744·0-744·3	Q16-Q17
9	Anomalies of the face and neck		744·4-744.9	Q18
10	***** Heart & circulatory system		745:747,425·3, (394:411*;414:417*;424·0:425·2*,425·4:426·9* used if recorded on death certificate)	Q20-Q28, I42·4
11	***** Heart		745-746	Q20-Q24
12	***** Circulatory system		747	Q25-Q28
13	Anomalies of the respiratory system	Anomalies of the respiratory system	748	Q30-Q34
14	***** Cleft Palate	Cleft Palate only	749·0	Q35

15	***** Cleft lip +/- Cleft palate	Cleft lip +/- Cleft palate	749·1-749·2	Q36-Q37
16	Anomalies of upper alimentary tract		750	Q38-Q40
17	Tracheo oesophogeal fistula	Tracheo oesophogeal fistula	750·3	Q39·1-Q39·3
18	Pyloric Stenosis		750·4	Q40·0
19	Other anomalies of the digestive system		751	Q41-Q45
20	Absence, atresia and stenosis of large intestine rectum and anal canal	Absence, atresia and stenosis of large intestine rectum and anal canal	751·2	Q42
21	Anomalies of genital organs	Anomalies of genital organs	752, 605, 778·6	Q50 - Q52, Q54-Q56
22	Hypospadias and epispadias		752.6	Q54,Q64·0
23	Anomalies of the urinary system	Anomalies of the urinary system	753	Q60-Q64
24	Renal agenesis and dysgenesis	Renal agenesis and dysgenesis	753	Q60
25	Certain musculo-skeletal anomalies	Musculoskeletal (all)	754	Q75·0-Q75.1, Q75·4-Q75·9,Q76·1-Q76·4, Q76·6-Q76·9, Q77, Q78, Q79·6-Q79·9
26	Congenital dislocation of the hip	Congenital dislocation of the hip	754·3	Q65·0 - Q65·6
27	Anomalies of the feet	Anomalies of the feet	754·5-754·7	Q66
28	Other anomalies of limb		755	Q69-Q74
29	Polydactyly	Polydactyly	755·0	Q69
30	Syndactyly	Syndactyly	755·1	Q70
31	Reduction deformaties of the limbs	Limb reductions	755·2-755·4	Q71-Q73
32	Other musculo-skeletal anomalies		756	Q75-Q79
33	Anomalies of diaphragm	Diaphragmatic defects	756·6	Q79·0-Q79·1

34	Exomphalos and Gastroschisis		756·7	Q79·2-Q79·3
35	Hernia of abdominal wall		550-553	K40-K46
36	Anomalies of skin and integument	Anomalies of skin and integument	757, 216, 228	Q80-Q84,D18,D22-D23
37	Chromosomal anomalies	Chromosomal anomalies	758	Q90-Q99
38	***** Down's Syndrome	Down's Syndrome	758·0	Q90
39	***** Trisomy 13	Trisomy 13	758·1	Q91·4- Q91·7
40	***** Trisomy 18	Trisomy 18	758·2	Q91·0-Q91·3
41	Multiple congenital anomalies		759	Q89
42	Other congenital anomalies		550-553, 243, 255, 270,277, 271, 282, 286, 524, 685, 771·0; 358*	E24-E27,E70-E74, E79-E80, E84-E85, D55-D58, D65-D68, E76, E88, K07, k40-K46, L05, E03·0, E03·1, P35·0

Supplementary Table 3. Additional detail to comply with the RECORD statement

	Item No.	STROBE items	Location in manuscript where items are reported	RECORD items	Response or location in manuscript where items are reported
**Title and abstract**					
	1	(a) Indicate the study's design with a commonly used term in the title or the abstract (b) Provide in the abstract an informative and balanced summary of what was done and what was found		RECORD 1.1: The type of data used should be specified in the title or abstract. When possible, the name of the databases used should be included.<br><br>RECORD 1.2: If applicable, the geographic region and timeframe within which the study took place should be reported in the title or abstract.<br><br>RECORD 1.3: If linkage between databases was conducted for the study, this should be clearly stated in the title or abstract.	Page 1<br><br>Page 1<br><br>Insufficient space in title or abstract
**Introduction**					
Background rationale	2	Explain the scientific background and rationale for the investigation being reported			Page 3
Objectives	3	State specific objectives, including any prespecified hypotheses			Page 3
**Methods**					
Study Design	4	Present key elements of study design early in the paper			Page 4
Setting	5	Describe the setting, locations, and relevant dates, including periods of recruitment, exposure, follow-up, and data collection			Page 4
Participants	6	*(a) Cohort study* - Give the eligibility criteria, and the sources and methods of selection of participants. Describe methods of follow-up<br>*Case-control study* - Give the eligibility criteria, and the sources and methods of case ascertainment and control selection. Give the rationale for the choice of cases and controls<br>*Cross-sectional study* - Give the eligibility criteria, and the sources and methods of selection of participants<br><br>*(b) Cohort study* - For matched studies, give matching criteria and number of exposed and unexposed<br>*Case-control study* - For matched studies, give matching criteria and the number of controls per case		RECORD 6.1: The methods of study population selection (such as codes or algorithms used to identify subjects) should be listed in detail. If this is not possible, an explanation should be provided.<br><br>RECORD 6.2: Any validation studies of the codes or algorithms used to select the population should be referenced. If validation was conducted for this study and not published elsewhere, detailed methods and results should be provided.<br><br>RECORD 6.3: If the study involved linkage of databases, consider use of a flow diagram or other graphical display to demonstrate the data linkage	Delivery records for all deliveries in select Scottish hospitals (list provided by AFFIRM) for years 2014-16. Established national dataset SMR02: http://www.isdscotland.org/Health-topics/Maternity-and-births/<br><br>No additional validation performed for this study.<br><br>NSS datasets are linked to the CHI database using probability matching.

			process, including the number of individuals with linked data at each stage.	The CHI number is then used as linkage key between datasets. For this study linkage between datasets was used for the Scottish data only. And only in relation to creation of a congenital abnormality flag. This used mother CHI from SMR02 to link to NRS Births, which then provides a link to child CHI. This child CHI was used to interrogate (1)SBR (2)NRS stillbirths (3)NRS infant deaths for congen flag. List of ICD10 codes attached to email (congen_list_August09.xls)
Variables	7	Clearly define all outcomes, exposures, predictors, potential confounders, and effect modifiers. Give diagnostic criteria, if applicable.	RECORD 7.1: A complete list of codes and algorithms used to classify exposures, outcomes, confounders, and effect modifiers should be provided. If these cannot be reported, an explanation should be provided.	File congen_list_August09.xls shown above (Supplementary Table 2) with all ICD10 codes used when creating congenital abnormality flag. All other data as recoded in national datasets
Data sources/ measurement	8	For each variable of interest, give sources of data and details of methods of assessment (measurement).<br>Describe comparability of assessment methods if there is more than one group		Data sources were hospital records for each data item. No attempt was made to standardise data collection (eg maternal age, stillbirth) between different groups as we used routinely collected data. However, the data items are in common use, and we do not anticipate any major difference in measurement between hospitals.
Bias	9	Describe any efforts to address potential sources of bias		Page 5
Study size	10	Explain how the study size was arrived at		Supplementary appendix 2 (statistical analysis plan)
Quantitative variables	11	Explain how quantitative variables were handled in the analyses. If applicable, describe which groupings were chosen, and why		Supplementary appendix 2 (statistical analysis plan)
Statistical methods	12	(a) Describe all statistical methods, including those used to control for confounding<br>(b) Describe any methods used to examine subgroups and interactions<br>(c) Explain how missing data were addressed<br>(d) *Cohort study* - If applicable, explain how loss to follow-up was addressed<br>*Case-control study* - If applicable, explain how matching of cases and controls was addressed<br>*Cross-sectional study* - If applicable, describe analytical methods taking account of sampling strategy<br>(e) Describe any sensitivity analyses		Supplementary appendix 2 (statistical analysis plan)
Data access and cleaning methods		..	RECORD 12.1: Authors should describe the extent to which the investigators had access to the database population used to create the study population.	Data was linked in the safe haven: most authors did not have access to the raw data.

			RECORD 12.2: Authors should provide information on the data cleaning methods used in the study.	Non-Scottish Data submitted from individual hospitals per year were subject to minor cleaning. This included formatting of different excel/csv files to standardise to aide joining all files together into one analysis file covering all locations and years. No data was altered. Any data that appeared incorrect (eg. Out of expected code range) were left until statistical analysis.<br>During statistical analysis unfeasible out of range values were set to missing. We also converted to a common measurement unit for the parameters maternal height and weight
Linkage		..	RECORD 12.3: State whether the study included person-level, institutional-level, or other data linkage across two or more databases. The methods of linkage and methods of linkage quality evaluation should be provided.	Data presented at individual level with enough information available to aggregate to hospital of delivery, Health board, country of delivery & SIMD decile.<br><br>Further references on data quality and methodology is below.<br><br>(A)Health Bull (Edinb). 1993 Mar;51(2):72-9. The **Scottish Record Linkage System**. Kendrick S(1), Clarke J. Author information: (1)Information and Statistics Division, Scottish Health Service, Edinburgh.<br><br>and<br><br>(B)http://www.isdscotland.org/Products-and-services/Edris/Docs/20150421-Linking-ScotXed-Data.pdf
**Results**				
Participants	13	(a) Report the numbers of individuals at each stage of the study (*e.g.*, numbers potentially eligible, examined for eligibility, confirmed eligible, included in the study, completing follow-up, and analysed)<br>(b) Give reasons for non-participation at each stage.<br>(c) Consider use of a flow diagram	RECORD 13.1: Describe in detail the selection of the persons included in the study (*i.e.*, study population selection) including filtering based on data quality, data availability and linkage. The selection of included persons can be described in the text and/or by means of the study flow diagram.	Consort diagram (Figure 1)
Descriptive data	14	(a) Give characteristics of study participants (*e.g.*, demographic, clinical, social) and information on exposures and potential confounders		Tables

		(b) Indicate the number of participants with missing data for each variable of interest<br>(c) *Cohort study* - summarise follow-up time (*e.g.*, average and total amount)		
Outcome data	15	*Cohort study* - Report numbers of outcome events or summary measures over time<br>*Case-control study* - Report numbers in each exposure category, or summary measures of exposure<br>*Cross-sectional study* - Report numbers of outcome events or summary measures		Tables
Main results	16	(a) Give unadjusted estimates and, if applicable, confounder-adjusted estimates and their precision (e.g., 95% confidence interval). Make clear which confounders were adjusted for and why they were included<br>(b) Report category boundaries when continuous variables were categorized<br>(c) If relevant, consider translating estimates of relative risk into absolute risk for a meaningful time period		Tables<br>Tables<br>Tables
Other analyses	17	Report other analyses done—e.g., analyses of subgroups and interactions, and sensitivity analyses		Tables
**Discussion**				
Key results	18	Summarise key results with reference to study objectives		Page 5 and 6
Limitations	19	Discuss limitations of the study, taking into account sources of potential bias or imprecision. Discuss both direction and magnitude of any potential bias	RECORD 19.1: Discuss the implications of using data that were not created or collected to answer the specific research question(s). Include discussion of misclassification bias, unmeasured confounding, missing data, and changing eligibility over time, as they pertain to the study being reported.	Page 6 and 7
Interpretation	20	Give a cautious overall interpretation of results considering objectives, limitations, multiplicity of analyses, results from similar studies, and other relevant evidence		Page 7
Generalisability	21	Discuss the generalisability (external validity) of the study results		Page 7
**Other Information**				
Funding	22	Give the source of funding and the role of the funders for the present study and, if applicable, for the original study on which the present article is based		Page 8
Accessibility of protocol, raw data, and programming code		..	RECORD 22.1: Authors should provide information on how to access any supplemental information such as the study protocol, raw data, or programming code.	Supplementary appendix 2

*Reference: Benchimol EI, Smeeth L, Guttmann A, Harron K, Moher D, Petersen I, Sørensen HT, von Elm E, Langan SM, the RECORD Working Committee. The REporting of studies Conducted using Observational Routinely-collected health Data (RECORD) Statement. PLoS Medicine 2015.

*Checklist is protected under Creative Commons Attribution (CC BY) license

Supplementary Table 4: Relative risks: Stillbirth and perinatal mortality; pregnancy and baby secondary outcomes

	Relative risk (95% CI)	
	**Intention to treat**	**On-treatment**
Live births	Control: 156963	Control: 251251
Stillbirth primary outcome*		
≥ 24 weeks gestation	0.90 (0.75, 1.07)	0.88 (0.76, 1.02)
Stillbirth secondary outcomes		
≥ 22 weeks gestation*	0.89 (0.75, 1.07)	0.89 (0.77, 1.02)
≥ 28 weeks gestation	0.97 (0.79, 1.18)	0.92 (0.79, 1.08)
≥ 37 weeks gestation	0.94 (0.69, 1.26)	1.05 (0.85, 1.29)
≥ 22 weeks gestation in normally formed infants*	0.98 (0.80, 1.21)	0.86 (0.71, 1.05)
≥ 24 weeks gestation in normally formed infants*	0.98 (0.79, 1.21)	0.86 (0.70, 1.05)
≥ 28 weeks gestation in normally formed infants	1.02 (0.80, 1.29)	0.95 (0.76, 1.18)
≥ 37 weeks gestation in normally formed infants	0.88 (0.61, 1.24)	1.00 (0.74, 1.35)
Perinatal mortality	0.98 (0.83, 1.17)	0.95 (0.81,1.12)
Preterm pregnancy	1.05 (1.00, 1.09)	1.00 (0.96, 1.04)
C-section	1.06 (1.04, 1.09)	1.10 (1.08,1.13)
Induction at ≥ 39 weeks	1.05 (1.03, 1.07)	0.91 (0.89,0.92)
Induction	1.03 (1.01, 1.05)	0.88 (0.86, 0.90)
Elective delivery	1.02 (1.00, 1.03)	0.94 (0.93, 0.95)
Elective delivery at ≥ 39 weeks gestation	1.03 (1.01, 1.05)	0.92 (0.91, 0.94)
Spontaneous vaginal delivery	0.96 (0.95, 0.97)	0.94 (0.92, 0.95)
Admitted to neonatal unit	1.01 (0.97, 1.06)	1.04 (1.00. 1.08)
Admitted to neonatal unit for > 48h	1.11 (1.05, 1.17)	0.81 (0.77, 0.86)
Admitted to neonatal unit at ≥ 37 weeks gestation	0.95 (0.90, 1.01)	1.03 (0.98, 1.08)
SGA delivered ≥ 40 weeks gestation	0.87 (0.79, 0.95)	0.94 (0.86, 1.02)
Preterm baby	1.04 (1.00, 1.09)	1.00 (0.96, 1.04)

C-section, caesarean section; SGA small for gestational age (≤ 10th centile); CI, confidence interval. Relative risk figures are for intervention versus control and are calculated from the adjusted odds ratios (Table 2 and Table 3) for a woman of average risk. All live births were included in the denominator, regardless of estimated gestation or weight. *If estimated gestation was missing, babies weighing 500g or more at delivery were included in the numerator for stillbirths at 22 or 24 weeks gestation or more, but not for stillbirths at 28 or 37 weeks gestation.

Supplementary Table 5. Population characteristics and mother and baby endpoints by intervention period (on treatment analysis)

		Intervention N=141480	Control N=252377	Washout N=15318	Overall N=409175
Maternal Age (years)		30.4 ± 5.8	29.9 ± 5.7	30.4 ± 5.8	30.1 ± 5.8
Ethnicity	White	113413 (80.2)	179402 (74.4)	11841 (77.4)	304656 (76.6)
	Mixed	2138 (1.5)	4039 (1.7)	267 (1.7)	6444 (1.6)
	Asian	7795 (5.5)	19097 (7.9)	888 (5.8)	27780 (7.0)
	Black African	2341 (1.7)	4406 (1.8)	270 (1.8)	7017 (1.8)
	Black Caribbean	1069 (0.8)	2978 (1.2)	104 (0.7)	4151 (1.0)
	Arab/Other ethnic group	2280 (1.6)	4316 (1.8)	295 (1.9)	6891 (1.7)
BMI description	Underweight	3388 (2.6)	5477 (2.8)	373 (2.5)	9238 (2.7)
	Normal	62873 (48.6)	92777 (47.5)	7310 (49.6)	162960 (48.0)
	Overweight	36087 (27.9)	55057 (28.2)	4166 (28.3)	95310 (28.1)
	Obese	26926 (20.8)	41937 (21.5)	2877 (19.5)	71740 (21.2)
Smoking during pregnancy		18510 (13.2)	32692 (14.5)	2109 (14.0)	53311 (14.0)
Parity	0	53977 (39.7)	104421 (42.4)	5777 (39.0)	164175 (41.4)
	≥ 1	81833 (60.3)	141695 (57.6)	9020 (61.0)	232548 (58.6)
Decile of Deprivation	1 most deprived	4181 (8.2)	16809 (16.6)	476 (8.57)	21466 (13.6)
(Scottish Index of Multiple	2	5953 (11.6)	12667 (12.6)	649 (11.7)	19269 (12.2)
Deprivation, SIMD)	3	6001 (11.7)	10735 (10.7)	681 (12.3)	17417 (11.1)
	4	5671 (11.1)	9638 (9.6)	602 (10.8)	15911 (10.1)
	5	4898 (9.6)	9048 (9.0)	563 (10.1)	14509 (9.2)
	6	4666 (9.1)	8445 (8.4)	504 (9.1)	13615 (8.6)
	7	4793 (9.4)	8731 (8.7)	522 (9.4)	14046 (8.9)
	8	4565 (8.9)	8932 (8.9)	485 (8.7)	13982 (8.9)
	9	4775 (9.3)	8817 (8.8)	480 (8.7)	14072 (8.9)
	10 least deprived	5729 (11.2)	6910 (6.9)	590 (10.6)	13229 (8.4)
Estimated gestation (weeks)		39.0 ± 2.2	39 ± 2.3	39.1 ± 2.1	39.0 ± 2.2
Estimated gestation categories	≤24 weeks	374 (0.3)	717 (0.3)	29 (0.2)	1120 (0.3)
	>24 to ≤28 weeks	718 (0.5)	1232 (0.5)	72 (0.5)	2022 (0.5)
	>28 to ≤32 weeks	1412 (1.0)	2686 (1.1)	160 (1.1)	4258 (1.1)
	>32 to ≤34 weeks	2007 (1.4)	3604 (1.5)	232 (1.5)	5843 (1.4)
	>34 to ≤37 weeks	15750 (11.2)	27609 (11.1)	1565 (10.2)	44924 (11.1)
	>37 to ≤39 weeks	55741 (39.5)	97612 (39.3)	6030 (39.4)	159383 (39.4)
	>39 to ≤41weeks	62303 (44.1)	109189 (44.0)	6894 (45.0)	178386 (44.1)

	>41 weeks	2940 (2.1)	5582 (2.3)	325 (2.1)	8847 (2.2)
Number of births this pregnancy (%)	1	139132 (98.3)	248222 (98.4)	15073 (98.4)	402427 (98.4)
	>1	2348 (1.7)	4155 (1.67)	245 (1.6)	6748 (1.7)
Mode of delivery	Spontaneous vaginal delivery	82566 (58.4)	147120 (58.3)	9042 (59.0)	238728 (58.4)
	Assisted vaginal delivery	18120 (12.8)	29526 (11.7)	1870 (12.2)	49516 (12.1)
	Elective C-section	18606 (13.2)	31316 (12.4)	1995 (13.0)	51917 (12.7)
	Emergency C-section	21444 (15.2)	35532 (14.1)	2297 (15.0)	59273 (14.5)
	Other or unspecified	688 (0.5)	8844 (3.5)	112 (0.7)	9644 (2.4)
Induction of labour	NONE	80990 (64.4)	125542 (57.5)	9343 (67.7)	215875 (60.3)
	ARM or ARM & oxytocics	7494 (6.0)	8622 (4.0)	841 (6.1)	16957 (4.7)
	Oxytocics	2064 (1.6)	2548 (1.2)	258 (1.9)	4870 (1.4)
	Any prostaglandin	23776 (18.9)	31100 (14.3)	2279 (16.5)	57155 (16.0)
	Other or unknown	11392 (9.1)	50445 (23.1)	1090 (7.9)	62927 (17.6)
Any fetal abnormality		6275 (4.8)	10016 (4.6)	579 (3.8)	16870 (4.6)
Baby Sex	Male	73561(51.1)	131240(51.1)	7973(51.2)	212774(51.1)
	Female	70234(48.8)	125134(48.8)	7594(48.8)	202962(48.8)
	Not determined	44(0.0)	263(0.1)	9(0.1)	316(0.1)
Birthweight categories	≤ 2500g	10686 (7.4)	20561 (8.0)	1100 (7.1)	32347 (7.8)
	> 2500g to < 3500g	68599 (47.8)	127756 (49.9)	7516 (48.4)	203871 (49.1)
	> 3500g to < 4000g	45334 (31.6)	77046 (30.1)	4834 (31.1)	127214 (30.7)
	≥ 4000g	18918 (13.2)	30497 (11.9)	2096 (13.5)	51511 (12.4)
Birthweight (g)		3373.9 ± 622.8	3338.7 ± 625.9	3375.6 ± 620.4	3352.2 ± 624.8
Birthweight centiles	≤ 10%	6391 (4.5)	13429 (5.4)	693 (4.5)	20513 (5.0)
	>10% to 90%	104013 (72.8)	185225 (73.9)	11315 (73.0)	300553 (73.5)
	≥ 90%	32522 (22.8)	52032 (20.8)	3489 (22.5)	88043 (21.5)
Apgar at 5 min	0	201 (0.2)	218 (0.1)	19 (0.1)	438 (0.1)
	1	119 (0.1)	205 (0.1)	16 (0.1)	340 (0.1)
	2	123 (0.1)	184 (0.1)	9 (0.1)	316 (0.1)
	3	167 (0.1)	230 (0.1)	18 (0.1)	415 (0.1)
	4	266 (0.2)	363 (0.2)	29 (0.2)	658 (0.2)
	5	480 (0.4)	837 (0.3)	61 (0.4)	1378 (0.4)
	6	1000 (0.7)	1703 (0.7)	103 (0.7)	2806 (0.7)
	7	1568 (1.2)	2893 (1.2)	176 (1.2)	4637 (1.2)
	8	3198 (2.4)	5841 (2.4)	369 (2.5)	9408 (2.4)
	9	82906 (61.3)	133809 (54.5)	8251 (55.8)	224966 (56.9)
	10	45310 (33.5)	99103 (40.4)	5747 (38.8)	150160 (38.0)

Apgar at 5 min < 4	610 (0.45)	837 (0.34)	62 (0.42)	1509 (0.38)
Apgar at 5 min < 7	2356 (1.74)	3740 (1.52)	255 (1.72)	6351 (1.61)
Resuscitation used	8679 (7.75)	13834 (7.06)	883 (7.40)	23396 (7.31)

Data in table are frequency (%), except for maternal age, estimated gestation and birthweight which are mean ± SD. Denominator for characteristics up to and including fetal abnormality is number of mothers, from sex of baby and onwards it is number of babies. For baby data, N=160465 (control group), 231813 (intervention group), 24022 (washout) and 416300 (overall).

C-section, caesarean section; BMI, body mass index. BMI categorisation defined as follows: underweight <18.5kgm-2, normal ≥ 18.5 and < 25, overweight ≥ 25 and <30, Obese ≥ 30. Number (%age) of values that were missing: maternal age 916 (0.2%); ethnicity 52236 (12.8%); BMI 69927 (17.1%); smoking during pregnancy 28592 (7.0%); parity 12452 (3.0%); decile of deprivation 378 (of 157,894 Scotland participants, 0.2%); estimated gestation 4373 (1.1%); mode of delivery 97 (<0.1%); induction of labour 51391 (12.6%); any fetal abnormality 44269 (10.8%); sex of baby 248 (0.1%); birthweight 1357 (0.3%); birthweight centile 7191 (1.7%); Apgar at 5 min 20778 (5.0%); resuscitation used 96385 (23.2%).
SIMD (Scottish Index of Multiple Deprivation 2016) for participants in Scotland only, based on ranking of small geographical areas from most deprived (ranked 1) to least deprived (ranked 6,976), see http://www.gov.scot/Topics/Statistics/SIMD for more details.

Supplementary Table 6. Stillbirth and perinatal mortality for on treatment analysis

	Intervention N=141480	Control N=252377	Adjusted odds ratio (95% CI)	p-value	Absolute effect (95% CI) per 10,000 pregnancies
Live births	140888	251251			
Stillbirth primary outcome*					
≥ 24 weeks gestation	550 (3.90)	1084 (4.31)	0.88 (0.76,1.02)	0.088	5 fewer (10 fewer to 1 more)
Stillbirth secondary outcomes					
≥ 22 weeks gestation*	561 (3.98)	1100 (4.38)	0.88 (0.76,1.02)	0.100	5 fewer (10 fewer to 1 more)
≥ 28 weeks gestation	409 (2.90)	803 (3.20)	0.92 (0.79,1.08)	0.308	2 fewer (7 fewer to 2 more)
≥ 37 weeks gestation	179 (1.27)	335 (1.33)	1.05 (0.85,1.29)	0.679	1 more (2 fewer to 4 more)
≥ 22 weeks gestation in normally formed infants*	473 (3.36)	853 (3.40)	0.86 (0.71,1.05)	0.136	5 fewer (10 fewer to 2 more)
≥ 24 weeks gestation in normally formed infants*	466 (3.31)	841 (3.35)	0.86 (0.70,1.05)	0.129	5 fewer (10 fewer to 2 more)
≥ 28 weeks gestation in normally formed infants	351 (2.49)	636 (2.53)	0.95 (0.76, 1.18)	0.640	1 fewer (6 fewer to 5 more)
≥ 37 weeks gestation in normally formed infants	154 (1.09)	277 (1.10)	1.00 (0.74,1.35)	0.998	0 fewer (3 fewer to 4 more)
Perinatal mortality	753 (6.45)	1454 (6.44)	0.95 (0.81,1.12)	0.559	3 fewer (12 fewer to 8 more)

Frequency data presented as N (rate per 1000 live births), except for perinatal mortality, which is N (rate per 1000 births). CI, confidence interval. Odds ratios are adjusted for maternal age, number of babies in the pregnancy and year. Number (%age) of values that were missing: stillbirth ≥ 24 weeks gestation 82 (0·02%) ; stillbirth ≥ 22 weeks gestation 57 (0·01%); stillbirth ≥ 28 weeks gestation 503 (0·13%); stillbirth ≥ 37 weeks gestation 1184 (0·31%); stillbirth ≥ 22 weeks gestation in normally formed infants 378 (0·10%); stillbirth ≥ 24 weeks gestation in normally formed infants 395 (0·10%); stillbirth ≥ 28 weeks gestation in normally formed infants 720 (0·19%); stillbirth ≥ 37 weeks gestation in normally formed infants 1266 (0·33%); perinatal mortality 50828 (13.2%). All live births were included in the denominator, regardless of estimated gestation or weight. For perinatal mortality, the denominator is number of babies: N=225612 (control) and N=116716 (intervention).
*If estimated gestation was missing, babies weighing 500g or more at delivery were included in the numerator for stillbirths at 22 or 24 weeks gestation or more, but not for stillbirths at 28 or 37 weeks gestation.

Supplementary Table 7 Pregnancy and baby secondary outcomes for on treatment analysis

	Intervention N=141480	Control N= 252377	Adjusted odds ratio (95% CI)	p-value
Preterm birth	10330 (7.3)	18972 (7.6)	1.00 (0.95, 1.04)	0.933
Caesarean section	40050 (28.3)	66848 (26.5)	1.15 (1.12, 1.18)	<0.001
Induction 39 weeks or more	31402 (33.6)	62464 (40.0)	0.85 (0.83, 0.88)	<0.001
Induction	44726 (34.7)	92715 (41.7)	0.81 (0.78, 0.84)	<0.001
Elective Delivery	62945 (48.8)	120900 (54.3)	0.88 (0.86, 0.90)	<0.001
Elective Delivery ≥ 39 weeks	43413 (46.5)	80876 (51.8)	0.85 (0.83, 0.88)	<0.001
Spontaneous Vaginal Delivery	82566 (58.4)	147120 (58.3)	0.86 (0.84, 0.88)	<0.001
Admitted to neonatal unit	10733 (9.4)	22392 (10.5)	1.05 (1.00, 1.10)	0.037
Admitted to neonatal unit more than 48h	6180 (5.4)	15206 (7.2)	0.80 (0.76, 0.85)	<0.001
Admitted to neonatal unit at ≥ 37 weeks gestation	5896 (5.7)	12481 (6.5)	1.03 (0.97, 1.09)	0.292
SGA delivered ≥ 40 weeks gestation	2120 (1.5)	4636 (1.9)	0.93 (0.86, 1.02)	0.153
Preterm baby	11810 (8.2)	21516 (8.5)	1.00 (0.95, 1.04)	0.844

Summary statistics in table are N (%). C-section, caesarean section; SGA, small for gestational age ≤ 10th centile, CI, confidence interval. Odds ratios are adjusted for maternal age, number of babies in the pregnancy and year. Number (%age) of values that were missing: preterm pregnancy 4307 (1·1%); C-section 95 (0·02%); induction at ≥ 39 weeks 140930 (36.6%); induction of labour 41183 (10·7%); elective delivery 41239 (10·7%); elective delivery at ≥ 39 weeks gestation 140945 (36·6%); spontaneous vaginal delivery 95 (0·02%); admitted to neonatal unit 72405 (18·5%); admitted to neonatal unit for >48h 72405 (18·5%); admitted to neonatal unit at ≥ 37 weeks gestation 103029 (26·3%); SGA delivered ≥ 40 weeks gestation 6963 (1·8%); preterm baby 4372 (1·1%).
Absolute effect sizes are per 10,000 babies for outcomes of neonatal unit admission, born small for gestational age or preterm baby.

Table 8 Pregnancy and baby secondary outcomes for on treatment analysis

	Adjusted mean difference (95% CI)	p-value
Estimated gestation (weeks)	-0·03 (-0·05, -0·00)	0·025
Estimated gestation (weeks) for inductions only	-0·06 (-0·10, -0·02)	0·007
Birthweight centile	0·16 (-0·17, 0·48)	0·341

Adjusted mean difference (intervention – control) is calculated after adjusting for maternal age, number of babies in the pregnancy and year. CI, confidence interval. Missing values: estimated gestation 4362 (1·1%); estimated gestation, inductions only 3591 (2·7%); birthweight centile 6867 (1·7%).

Table 9
Early neonatal, late neonatal and post neonatal deaths, per protocol analysis.

Outcome	Intervention group, N = 227,860<br>n / denominator (rate per 1000)	Control N = 157,692<br>n / denominator (rate per 1000)
Early neonatal death < 7 days of life	457 / 200,339[a] (2.28)	369 / 135,591[b] (2.72)
Late neonatal death (7 days - 28 days of life)	78 / 196,478[c] (0.40)	59 / 130,143[d] (0.45)
Post neonatal death (28 days to 1 year)	74/ 201,607[e] (0.37)	24 / 126,770[f] (0.10)

n missing = a - 27461, b-22101, c – 31382, d – 27549, e – 26253, f- 30922. Note, these outcomes were not included in the pre-specified statistical analysis plan, but have been included at the request of a reviewer during the submission of this manuscript.

Supplementary Appendix 1. Sites and investigators

England

Blackpool Women's Hospital, Blackpool Teaching Hospitals NHS Foundation Trust
Mr Johnson Amu, Consultant Obstetrician and Gynaecologist; Carolyn (Jane) Brooks, Research Midwife

Women and Children's Research Team, Royal Preston Hospital, Lancashire Teaching Hospitals NHS Trust
Gillian Byrne, Information Technology Midwife; Angela Philipson, Research Midwife; Miss Fatimah Soydemir, Consultant Obstetrician and Gynaecologist

Leeds General Infirmary, Leeds Teaching Hospitals NHS Trust
Mr Nigel Simpson, Consultant Obstetrician and Gynaecologist; Viv Dolby, Research Midwife, Tracey Glanville, Consultant; Sharon Nettleton, Research Midwife

St Mary's Hospital, Manchester, Manchester University NHS Foundation Trust
Prof Alex Heazell, Consultant Obstetrician and Gynaecologist; Heather Glossop, Research Midwife

St George's Hospital, London, St George's Healthcare NHS Trust
Miss Asma Khalil, Consultant Obstetrician and Gynaecologist; Eleanor Lindahl, Research Midwife; Yvonne Muwalo, Research Midwife; Mr Basky Thilaganathan, Consultant Obstetrician and Gynaecologist

Stepping Hill Hospital, Stockport, Stockport NHS Foundation Trust
Janet Cotton, Clinical Midwifery Manager; Julie Estcourt, Head of Midwifery; Julie Grindey, Research Midwife; Miss Rachel Owen, Consultant Obstetrician and Gynaecologist; Miss Madhavi Pureti, Consultant Obstetrician and Gynaecologist

St Richard's Hospital, Chichester, Western Sussex Hospitals NHS Foundation Trust
Michelle Lane, Research Midwife; Emma Meadows, Research Midwife; Miss Sophia Stone, Consultant Obstetrician and Gynaecologist

Worthing and Southlands Hospital, Chichester, Western Sussex Hospitals NHS Foundation Trust
Vivienne Cannons, Research Midwife; Miss Rahila Khan, Consultant Obstetrician and Gynaecologist

Royal Albert Edward Hospital, Wigan, Wrightington, Wigan and Leigh NHS Foundation Trust
Dr Amanda Bellis, Consultant Obstetrician and Gynaecologist; Linzi Heaton, Clinical Trials Administrator; Tracey Taylor, Clinical Research Nurse; Mr Amit Verma, Consultant Obstetrician and Gynaecologist

Ireland

Royal Jubilee Maternity Hospital, Belfast Health and Social Care Trust
Sylvia Craig, Research Midwife; Dr Alyson Hunter, Consultant Obstetrician and Gynaecologist; Brenda Kelly, Head of Midwifery; Kathleen McCandless, Midwifery Sister; Mr Arthur McNally, Consultant Obstetrician and Gynaecologist; Helen Rice, Research Midwife;

National Maternity Hospital, Dublin
Professor Fionnuala McAuliffe, Consultant Obstetrician and Gynaecologist; Prof Mary Higgins, Consultant Obstetrician and Gynaecologist

Rotunda Hospital, Dublin
Prof Fionnuala Breathnach, Associate Professor, Obstetrics and Gynaecology; Prof Michael Geary, Consultant Obstetrician and Gynaecologist; Prof Fergal Malone, Consultant Obstetrician and Gynaecologist; Dr Elizabeth Tully, Research Manager

Scotland

Aberdeen Maternity Hospital, NHS Grampian
Dr Asha Shetty, Consultant Obstetrician and Gynaecologist

Ayrshire Maternity Unit, Crosshouse Hospital, NHS Ayrshire and Arran
Dr Jane Ramsay, Clinical Director for Obstetrics

Borders General, NHS Borders
Nicky Berry, Head of Midwifery;;
Dr Imogen Hayward, Obstetric Anaesthetist; Susie McFadzen, Research Midwife; Dr Brian Magowan, Consultant Obstetrician and Gynaecologist

Cresswell Maternity Unit, Dumfries, NHS Dumfries and Galloway
Nicola Harper, Senior Charge Midwife; Dr Stephen Wisdom, Consultant Obstetrician and Gynaecologist

Victoria Hospital, Kirkcaldy, NHS Fife
Dr Jennifer Boyd, Consultant Obstetrician and Gynaecologist; ; Dr Helen Russell, Consultant Obstetrician and Gynaecologist; Dr Graham Tydeman, Consultant Obstetrician and Gynaecologist

Forth Valley Hospital, Stirling, NHS Forth Valley
Dr Klara Ekevall, Consultant Obstetrician and Gynaecologist; Dr Shahzya Huda, Consultant Obstetrician and Gynaecologist; Dr Nicola Miller, Consultant Obstetrician and Gynaecologist; Mairi Whitfield, Senior Midwife

Dr Gray's Hospital, Elgin, NHS Grampian
Prof Grant Cumming, Consultant Obstetrician and Gynaecologist

Wishaw General Hospital, NHS Lanarkshire
Wendy Drysdale, Senior Midwife; Dr Dina McLellan, Consultant Obstetrician and Gynaecologist

St John's Hospital, Livingston, NHS Lothian
Katy Court, Acting Clinical Manager, Midwifery Services: Dr Sarah Court, Consultant Obstetrician and Gynaecologist

Simpson Centre for Reproductive Health, RIE, NHS Lothian
Dr Corinne Love, Consultant Obstetrician and Gynaecologist; Karen Nattan, MCQIC champion; Sue Shade, Senior Charge Midwife

Wales

Betsi Cadwaladr University Health Board, Wales
Mr Phil Banfield, Consultant Obstetrician and Gynaecologist; Annette Bolger, Research Officer; Sarah Davies, Research Midwife; Lynda Sackett, Research Midwife; Jayne Jones, Head of Research Delivery

Nevill Hall Hospital, Aneurin Bevan University Health Board, Wales
Mrs Anju Kumar, Clinical Director; Miss Delyth Rich, Consultant Obstetrician & Gynaecologist

Royal Gwent Hospital, Aneurin Bevan University Health Board, Wales
Deb Jackson, Head of Midwifery; Emma Mills, Research Midwife; Miss Manju Nair, Consultant Obstetrician & Gynaecologist;
